# Emergency medical technicians' experiences with unplanned births outside institutions: A qualitative interview study

**DOI:** 10.1002/nop2.354

**Published:** 2019-07-31

**Authors:** Hanne Vagle, Gunn Terese Haukeland, Bente Dahl, Vigdis Aasheim, Eline Skirnisdottir Vik

**Affiliations:** ^1^ Faculty of Health and Social Sciences Western Norway University of Applied Sciences Bergen Norway; ^2^ Centre for Women's, Family and Child Health, Faculty of Health and Social Sciences University of South‐Eastern Norway Kongsberg Norway; ^3^ Department of Global Public Health and Primary Care University of Bergen Bergen Norway

**Keywords:** born before arrival, born unplanned outside institutions, emergency medical technician, experience, midwifery, out‐of‐hospital birth, paramedic, patient safety, pre‐hospital birth, specialist ambulance nurse

## Abstract

**Aim:**

To explore emergency medical technicians' experiences with unplanned births outside institutions.

**Design:**

A qualitative interview study.

**Methods:**

Individual semi‐structured interviews with 12 emergency medical technicians in Norway. Systematic text condensation was used to analyse the data material.

**Results:**

Analysis showed that there is a mismatch between society's expectations about emergency medical technicians and the reality they encounter in out‐of‐hospital maternity care, that emergency medical technicians experience a general lack of training in caring for labouring women and that poor communication with other health professions challenges patient safety. The participants expressed how they do their best in caring for both mother and child, in spite of a lack of education, training and competence in assisting labouring women.

## 1 INTRODUCTION

The number of children born unplanned outside institutions is increasing in many countries such as Norway, Finland and Ireland (Engjom, Morken, Norheim, & Klungsøyr, 2014; Gunnarsson, Smárason, Skogvoll, & Fasting, 2014; Hemminki, Heino, & Gissler, 2011; Unterscheider, Ma'ayeh, & Geary, 2011). In 2017, 344 infants were registered born unplanned outside institutions in Norway: this included 152 unplanned home births, 171 births en route to hospital, 16 births in unknown locations and five births in institutions without maternity care services (Medical Birth Registry of Norway, 2019). Unplanned births outside institutions include all accidental out‐of‐hospital births and are often dealt with by emergency medical technicians (EMTs) without assistance from midwives or physicians (Dietsch, Shackleton, Davies, Alston, & McLeod, 2010; McLelland, McKenna, Morgans, & Smith, 2018; McLelland, Morgans, & McKenna, 2013; Thornton & Dahlen, 2018). Reasons for the increasing number of out‐of‐hospital births have been attributed centralisation of care (Engjom, Morken, Høydahl, Norheim, & Klungsøyr, 2017) and midwives acting as gatekeepers of the labour ward (Eri, Blystad, Gjengedal, & Blaaka, 2010; Vik, Haukeland, & Dahl, 2016).

To ensure the safety of both mother and child, the World Health Organization (WHO) emphasizes the importance of skilled attendants assisting labouring women (World Health Organization, 2018). Little is known about EMTs' experiences with unplanned births outside institutions, but the phenomenon of women experiencing unplanned out‐of‐hospital births highlights concerns about the structure and process for triaging labouring women (Vik et al., 2016).

## 2 BACKGROUND

Unplanned out‐of‐hospital births are associated with an increased risk of infant mortality compared with births in obstetric institutions (Engjom et al., 2017; Jones et al., 2011; Nguyen, Lefevre, & Dreyfus, 2016). One Australian study reported that 11% of women attended by EMTs during labour gave birth before arriving at hospital; of these, 27% were recorded with antenatal and/or intrapartum complications, while 22% of the babies were recorded with an Apgar score lower than 7 (Flanagan, Lord, & Barnes, 2017). Risk factors associated with both maternal and neonatal outcomes in unplanned out‐of‐hospital births include maternal characteristics (young maternal age, low socio‐economic status, immigrant background, smoking), pregnancy‐related factors (multiparity, placental conditions, low birthweight, prematurity, infections, concealed pregnancy) and poor antenatal care (Boland et al., 2018; Engjom, Morken, Høydahl, Norheim, & Klungsøyr, 2018; Gunnarsson, Fasting, Skogvoll, Smárason, & Salvesen, 2017; Jones et al., 2011; Nguyen et al., 2016; Unterscheider et al., 2011).

According to Pettker and Grobman, the quality measures on patient safety include the following three factors: (a) the structure of maternity care; (b) the process for how we manage labouring women; and (c) the risk factors associated with the birth outcome (Pettker & Grobman, 2015). Given our knowledge of the risks related to giving birth unplanned out‐of‐hospital and the importance of being cared for intrapartum by a skilled professional, we set‐up this study to address the gap in the literature by reporting EMTs' experiences with unplanned births outside institutions. The findings in the study are discussed from a patient safety perspective. Based on the existing knowledge on unplanned births outside institutions, we posed the following research question: What experiences do EMTs' have with unplanned births outside institutions?

## 3 THE STUDY

### 3.1 Design

A qualitative interview‐based study.

### 3.2 Setting

In Norway, EMTs are the main caregivers for people in need of medical help in out‐of‐hospital emergency situations (Lovdata, 2005). Norway is a sparsely populated country where the structure of maternity care has been centralized in recent decades (Engjom et al., 2014). Travelling from home to hospital may include delays related to bad weather or ferry transport. The standard of the ambulance services is mandated by law (Lovdata, 2005), with two healthcare professionals staffing each ambulance (i.e. car/boat), one of whom must be a certified EMT. Additionally, the National Air Ambulance Services of Norway are staffed with both physicians and nurses (Lovdata, 2005). Today, EMTs in Norway are trained at high school level completed with 2 years apprenticeship and/or bachelor level including clinical placement periods (Paramedic, 180 ECTS) (The Norwegian Directorate for Education & Training, 2019). Even though Norway has one of the highest densities of physicians in Europe, the country still struggles to ensure social and geographical equity in access to health care (Ringard, Sagan, Sperre Saunes, & Lindahl, 2013). In 2008, 79% of Norwegian municipalities lacked a formalized accompaniment service in the case of out‐of‐hospital births (Egenberg, Puntervoll, & Øian, 2011).

### 3.3 Sample

We carried out semi‐structured individual interviews (Kvale, 2007) with 12 EMTs all of whom had professional experience with unplanned out‐of‐hospital births. The study participants included six male and six female EMTs. Their experiences ranged from 2–20 years in the ambulance services (mean: 11.5 years) and they were between 28–44 years of age. In addition to being certified EMTs, five had additional training in out‐of‐hospital care (Paramedic, 60 ECTS), while one was a certified nurse.

### 3.4 Data collection

Initially, we contacted two leaders of two different departments for emergency medical services in Western Norway. They invited employees to participate in the study by email. This strategy did not result in any participants. We widened the geographic area of interest to other departments in Western Norway. Further, we used acquaintances in our network to provide eligible EMTs with written information about the study. Contact information for potential participants was returned to the study group.

All interviews were conducted between September–November 2017 and took place in western parts of Norway, either in the home of the EMT or at their workplace. The interviews were recorded and lasted from 25–60 min (mean: 42 min). The information collected was rich and relevant for the study; thus, the limited number of participants was considered appropriate (Malterud, Siersma, & Guassora, 2016). Six different ambulance centres were represented in this study.

The interview guide consisted of five open questions. Initially, the EMTs were asked to share their experiences of assisting women in the case of unplanned out‐of‐hospital births, prompted by the question: “Could you please describe one time you helped deliver a baby while on duty?” They were encouraged to narrate freely and give rich descriptions of the birth, including all individuals involved in the situation. Secondly, they were asked if they could describe their own reflections after the mission. The third question encouraged them to describe their thoughts when going on a mission to help a labouring woman. In the fourth question, they were asked what they would change about today's practice if they were given the power to do so; and, finally, they were asked if there was anything they wanted to add to the interview based on their own knowledge and experiences of unplanned out‐of‐hospital births. The EMTs were only interrupted when clarification or elaboration was needed and there was no time limit to the interviews.

### 3.5 Data analysis

After transcribing the interviews, systematic text condensation (STC) was used to analyse the data material (Malterud, 2017). Systematic text condensation is a strategy for thematic cross‐case analysis and can be described in the following four steps: 1. The interviews were read to gain an overall impression. In this process, the following temporary themes emerged: midwife assisted transport service, practical training, lack of competence, handling complications, poor communication, decision‐making and EMTs' feelings. 2. Meaning units describing EMTs' experiences with unplanned out‐of‐hospital births were identified, marked and organized into three different code groups. 3. In each of the three code groups, another three subgroups were identified and meaning units in all subgroups were then summarized and condensed. 4. In the end, the text condensates formed the basis for our final analytic text. Quotations were used to elucidate the findings.

### 3.6 Rigour

One can argue that each qualitative study is unique and the diversity in qualitative research therefore challenges assessing the quality of each study (Rolfe, 2006). While there is no overarching set of criteria by which to judge the validity of qualitative research, we have included several methodical techniques to strengthen the quality of this study. These methodical techniques included taking field notes after each interview focusing on capturing emotions and preliminary interpretations of the content. Further, all interviews were conducted on a face‐to‐face basis at a location chosen by the participant (Guion, Diehl, & McDonald, 2001).

### 3.7 Reflexivity

The interviews were conducted by the first author as a part of her master's degree in midwifery. In addition to experiences and extensive knowledge about childbirth and labouring women, she has 10 years' experience as an EMT. Her practice as an EMT is in a Norwegian setting, but in a different district than the EMTs participating in this study. All authors are registered nurse midwives with experiences of antenatal, perinatal and postnatal care, including unplanned births outside institutions. The four co‐authors are involved in education on midwifery education programmes in Norway. One author has personal experience of unplanned birth outside institution.

## 4 RESULTS

Three main themes emerged from the analysis: 1. There is a mismatch between society's expectations about EMTs and the reality they meet in out‐of‐hospital maternity care; 2. EMTs experience a general lack of training in caring for labouring women; and 3. Practical challenges and poor communication with hospital midwives.

### 4.1 There is a mismatch between society's expectations about EMTs and the reality they encounter in out‐of‐hospital maternity care

The EMTs in this study described today's practice as vulnerable and stressed the importance of having an on‐call midwife available. Most EMTs explained that having an on‐call midwife was not standard practice in their area and those who did not have access to a community midwife suggested a midwife could be on‐call from the hospital. Emergency medical technicians who had access to a community midwife praised this service and the collaboration with the midwife. However, some discussed how they were not usually invited to assist the midwife, thus leaving the midwife alone with the labouring woman at the back of the ambulance. The EMTs revealed how they wished to be included alongside the midwife, underlining the fact that they had not necessarily seen a woman give birth before and were still expected to act quickly if an obstetric emergency occurred. The EMTs also said they knew that they might have to manage the situation without a skilled birth attendant in the ambulance. One EMT who had 7 years of practice talked about how she normally reacted when called to assist a woman in labour:

My immediate thought when we get a call like this is: ‘Where is the midwife?’ That is the first question I ask the call manager at the dispatch centre. (…) In the area where I work, we only have access to a midwife during the daytime; and, if we get hold of the midwife, it takes at least 30 minutes from the initial call until the midwife is actually in the car with us. (Interview 1)

Having access to a midwife was described as important to ensure patient safety and the EMTs described several examples of how a skilled birth attendant played a key role during obstetric emergencies, such as shoulder dystocia or breech position. One EMT described how they involved a local midwife when they experienced a stillbirth in a district without an organized midwife assisted transport service. The midwife assisted with the birth even though this was not her responsibility. Others referred to situations where they did ask for help, but ended up handling the situation themselves:

It was in the middle of the night and I remember it very well. At about four in the morning, we got a call from a woman in labour. Her waters had broken earlier in the evening and the patient had been sent home from the hospital after a check‐up. This was in the middle of nowhere, a two‐hour drive on bad roads and a ferry without a scheduled night service. The ferry was moored on the opposite side from where the woman lived. We drove past the local physicians' office and we called the physician on duty four times, but still the physician was not willing to make a house call. When we arrived, the woman was ready to push. (Interview 11)

Their lack of training in assisting labouring women was described as stressful, and one additional element brought up by several EMTs was that they did not have the knowledge, skills or necessary equipment to perform foetal heart rate monitoring, thus undermining how to handle the risk assessment. When participants spoke of their colleagues, most were described as professional in handling childbirth, while others were described as insecure and hesitant in relation to labouring women. It was highlighted as especially challenging working with inexperienced colleagues, as this EMT explained:

My colleague that day was very stressed (…) She was running around outside the ambulance confused, a little like a headless chicken (…) She completely lost her head. This could happen to anyone. But, when one denies it afterwards, it just makes it all the more difficult (…) If you are lucky, you have a midwife with you in the car and then it is never a problem. (Interview 2)

Being called on to assist a labouring woman can bring up mixed feelings, according to the EMTs. They revealed how they would be extremely focused; and, when the baby was born screaming, they felt relieved and overjoyed. The different feelings ranged from joy to fear, whereas some described a general feeling of adrenaline rushing though their body. Meanwhile, others said they might feel overwhelmed by a wave of different feelings. One EMT described her feelings in the following way:

The feeling you get when you are about to assist a labouring woman is excitement mixed up with fear… I am not one of those who dread these missions. Foremost, I think it is very exciting… birth is wonderful… and then I think to myself that it is good the mission came, because we seldom get to practise assisting labouring women. (Interview 10)

### 4.2 EMTs experience a general lack of training in caring for labouring women

The EMTs described how unplanned out‐of‐hospital births generally resulted in uncomplicated births. However, the positive outcomes are attributed to good fortune rather than the result of good practice. They explained how women who did not reach the hospital in time were at the mercy of nature and how they often had to depend mostly on the woman's antenatal record card for the information they needed to make appropriate decisions. On the antenatal record card, they read about the pregnancy and, if the woman was parous the EMTs hoped for an uncomplicated birth. They also revealed how some women were harder to communicate with than others, such as women from an immigrant background or who were focusing on their contractions and unable to answer questions. Occasionally, the only information available was that a woman with abdominal pain was in need of assistance. The information given to the EMTs was often incomplete, which affected them in their work. According to one EMT:

We did in fact experience the birth of twins once and that is unusual (…) It is a different story the day you know you are driving out to a woman with a high‐risk pregnancy. The mission is completely different if it is a breech position and you see the foetus hanging there with one foot out (…) Then you realize this is going to be a little bit more challenging than what you had hoped for. (Interview 3)

All EMTs expressed how they lacked training in assisting labouring women. They appreciated lectures on childbirth, but the extent of lectures offered varied a great deal. Some explained how a 1‐hr lecture was all they were offered, while those who received more than 1 hr revealed how the EMTs themselves had initiated a full‐day of lectures and training after a near‐miss or fatal experience in the local ambulance service. Several participants explained how their personal interest in childbirth played an important role in their practice, especially female EMTs who had given birth. One EMT indicated how his experiences with childbirth influenced his work:

I have my own personal experiences [with childbirth], of course (…) and I cling to these (…) together with the experiences of childbirth that I have from working in the ambulance services. Still, I cannot say that I am capable of handling the situation if something goes wrong during the labour. I am not. (Interview 5)

Personal experiences were described as valuable to the EMTs, as these helped them to identify gaps in knowledge and encourage them to learn more about childbirth. Where the EMTs acquired their personal experiences varied, ranging from giving birth themselves or accompanying a partner or a friend giving birth, to experiences of being on duty in the ambulance services. One EMT explained what inspired her to increase her knowledge of childbirth:

The main reason why I wanted to learn more about midwifery is that the patient expects you to be able to handle the situation. You arrive with an ambulance and they have called for help. But, as you know, in reality we know very little about labouring women. (Interview 11)

Overall, more experienced EMTs expressed less joy and more concern in relation to childbirth, as well as revealed how increased knowledge made them more aware that the lives of both mother and child can be at risk. With more experience, the EMTs also highlighted how they changed their practice over time and felt less stressed and calmer, as this experienced EMT stated:

After the baby is born I am calmer now than I used to be. I give the baby more time than I used to only a couple of years ago (…) Earlier, I was very active with the neonatal suction device, but this practice has changed (…) The babies are often blue when they are born. Now I know that this is normal and the births I have attended have had a positive effect on me [as an EMT]. (Interview 6)

### 4.3 Practical challenges and poor communication with hospital midwives

Most EMTs reflected on the challenge of deciding whether to rush the labouring woman to the hospital or allow her to give birth under calmer circumstances. Decision‐making was explained to be a great challenge on the road, and EMTs often had to be creative when making decisions. On the one hand, the EMTs said giving birth at home offered better opportunities than giving birth in a cramped ambulance, where they also had to make sure that both mother and baby were safely fastened, thus hindering uninterrupted skin‐to‐skin contact. On the other hand, giving birth in an ambulance meant they would be closer to the hospital in case of an emergency. Some EMTs had also experienced women who refused help from male EMTs or to even leave their home, resulting in a delay in departure to the hospital. One EMT discussed how they handled the situation when a baby was born premature at home:

We had to make room on the dining table and start resuscitating (…) we were actually resuscitating for a couple of hours (…) It was an unusual situation, because there was a little two‐ or three‐year‐old child running around in the living room (…) In the end we let the older child stay with her mother as the newborn child died. (Interview 9)

Further, the EMTs talked about how they would seek to increase their knowledge of complications by reading, watching videos or talking to colleagues. The EMTs emphasized that no one would wish for a woman to give birth unplanned out‐of‐hospital. Nonetheless, the EMTs were aware of the fact that there will always be women who do not reach hospital in time; and, as long as a midwifery service is not offered, it is crucial for EMTs to receive hands‐on training. One EMT put it like this:

It would be better for all if we had real training. We could practise on the delivery ward and assist childbirth there. If someone coached us, we could be more prepared [for childbirth] when we are on duty. We would never practise resuscitation by merely watching others. We would practise hands‐on ourselves and this should be the case for handling labouring women as well. (Interview 7)

The EMTs described difficulties in securing a clinical placement on delivery wards, having to depend instead on goodwill or personal acquaintances in the search for more practice. The clinical placement was often unpaid and scheduled in their spare time. The men in the study explained how they felt discriminated against, as their experience was that they were only allowed to observe and not assist. Several talked about how they felt it was inappropriate how medical students and student nurses were prioritized over EMTs, considering that EMTs are expected to deal with labouring women more frequently than physicians and nurses. One EMT expressed the strained relationship that EMTs have with midwives and labour wards in the following way:

They did not include us on the delivery ward. They did not let us in and they claimed it was unethical if they were to let us onto the ward. That was not good. In the neonatal intensive unit, however, they gave us an unlimited clinical placement (…) I know that a few ambulance workers have been on the delivery ward (…) It is almost like horse trading, you have to know someone to get in. It is difficult. So… we feel like… I mean… ambulance workers and midwives in this city… we really do have a problematic relationship. I do not know if anyone else has already told you this. (Interview 3)

Several EMTs described a troublesome relationship with the midwives at the hospital. Experienced EMTs, however, pointed out that this was not the case in all areas and that this relationship had improved over the years, with communication being better with recently trained midwives. Some described episodes where they were yelled at when arriving on the delivery ward. One said that midwives had accuse EMTs of driving too slowly, taking deliberate detours, while several EMTs stated how midwives complained if the EMTs had cut the umbilical cord on arrival. The EMTs explained that they knew the benefit of late clamping, but often had good reasons for cutting the cord, such as having to transport both mother and newborn in a safe way down narrow stairs in smaller buildings. The EMTs said that, in these situations, the midwives were often busier complaining than listening to the information handover, as this EMT clarified:

At the clinic, we were met by a midwife who did not want to talk with us. Another midwife on the same ward interfered and handled the information handover. The midwife who initially refused to talk was concerned with the fact that I had cut the umbilical cord, because this meant I had to sign a form before I left the hospital. (Interview 4)

Apart from on‐boat or in‐air services, most EMTs stated that two EMTs would be allocated to one ambulance. The weather could be bad and there could be long distances, bad roads or limited information about the labouring woman. Sometimes, only one of the EMTs was qualified to drive the car; and, in these cases, the driver was also the most experienced of the two. After a mission, the EMTs discussed how they left the patients at the hospital without further follow‐ups or information on how the assignment ended. The EMTs were expected to deal with any emergency as well as indicated how this affected them in both the short and the long term. One EMT mentioned going on sick leave after handling an assignment where the baby turned out to be in an undetected breech position:

I had so many roles. I was supposed to be her best friend, since her husband was not there… I was lying in her bed, I was supposed to help her as she gave birth to her child and then I delivered the child and it was then I had to start the resuscitation. (…) the baby died… there was no… there was no brain activity. They kept the baby alive until the family arrived (…) I went home after the assignment and I could not make myself go back to work after that. I was completely done, I just cried (…) And later… there was never any debrief… They had one at the hospital, but we were not invited. (Interview 8)

## 5 DISCUSSION

The findings in this study show how EMTs do their best to care for labouring women, despite their limited experience with childbirth. The EMTs meet different challenges in out‐of‐hospital care, ranging from a lack of training and education, practical challenges related to being outside a hospital and challenges related to poor communication with both labouring women and hospital midwives. The results are discussed below from a patient safety perspective (Pettker & Grobman, 2015).

Working in an emergency medicine service (EMS) obligates a broad understanding of a wide range of different scenarios (Wilson et al., 2015) and the EMTs in this study revealed how there is a mismatch between society's expectations of EMTs and EMTs' reality of actually having no or very little training in assisting labouring women. They are expected to care for the women and act quickly if an obstetric emergency occurs. The structure of maternity care is a key element when evaluating patient safety (Pettker & Grobman, 2015), while making a decision and acting quickly in the out‐of‐hospital period can greatly affect the outcome of an emergency (Wilson et al., 2015). The EMTs describe several scenarios where a midwife deals with a situation which they themselves do not feel competent to do so, including breech position and shoulder dystocia. Meanwhile, they also state how they are often left without assistance from midwives or physicians, especially in rural areas and after normal working hours, even though unplanned out‐of‐hospital births often occur at night (Ford & Pett, 2008). A degree of geographical lottery in the standard of emergency care is described by others (Lockey, 2009), and it is therefore understandable that the EMTs in this study advocate for an improved midwife assisted transport service. This finding is supported by the results from one Norwegian study highlighting the importance of skilled birth attendance and addressing the negative consequences of reduced access to institutions in districts (Engjom et al., 2017).

The process for how we support labouring women is a second key factor in evaluating patient safety (Pettker & Grobman, 2015). The EMTs in this study describe mixed feelings about assisting labouring women, a finding supported by a recent Swedish study (Persson, Engström, Burström, & Juuso, 2018). Another Swedish study explored strategies used by personnel in the emergency services, with the participants in this study confirming how childbirth in particular was a major stress factor in out‐of‐hospital care (Bohström, Carlström, & Sjöström, 2017). The EMTs in our study further discussed how they are met with scepticism from midwives when arriving at the hospital and how poor communication with midwives can be a challenge to patient safety, such as when the midwife shows no interest in the patient handover. Assisting labouring women can give EMTs a feeling of not being in control; hence, they might seek feedback on their efforts from hospital employees before leaving the hospital (Persson et al., 2018). A lack of feedback from receiving personnel, however, is recognized as a challenge in patient handovers between out‐of‐hospital and hospital staff (Wood, Crouch, Rowland, & Pope, 2015). Further, the EMTs also reveal that, in some cases, it is poor communication with labouring women that challenges patient safety, which can be explained by suboptimal maternal use of available care in the case of immigrant women (Unterscheider et al., 2011) or women with a concealed pregnancy (Gunnarsson et al., 2017). In the interests of improving care for these women, the EMTs underline the need for an increased focus on education and training for EMTs and how involvement by midwives or physicians when a woman gives birth unplanned out‐of‐hospital could be a valuable contribution to their future practice. Giving EMTs, the opportunity to receive scenario training on childbirth could also facilitate improvements in practice (Persson et al., 2018). To improve the understanding of each other's roles and responsibilities in assisting labouring women, interprofessional learning workshops involving both student midwives and student EMTs may be worth exploring (Feltham, Foster, Davidson, & Ralph, 2016).

The third factor associated with evaluating patient safety concerns the risks associated with the outcome of the birth. The EMTs in our study believe that their practice has improved with experience, parallel to their understanding of the risks these women and their unborn children are exposed to. They reveal how women giving birth unplanned out‐of‐hospital are at the mercy of nature and how EMTs are not necessarily included in debriefs after near‐miss cases or death. One Swedish study supports the low frequency of debrief sessions after stressful events in out‐of‐hospital care (Bohström et al., 2017), as well as describes how collegial support and discussions with colleagues can be a positive strategy for stress reducing after stressful events such as childbirth. The increased risk of infant morbidity and mortality, compared with births in obstetric institutions, is well documented (Engjom et al., 2017; Flanagan et al., 2017; Jones et al., 2011; Nguyen et al., 2016), even though one study from Finland reports no major perinatal or maternal cases in their sample (Pirneskoski, Peräjoki, Nuutila, & Kuisma, 2016). Maternal outcomes, such as postpartum haemorrhage and severe perineal tears, are however not fully understood (Ford & Pett, 2008; Unterscheider et al., 2011).

Most women experiencing unplanned out‐of‐hospital births in Norway are parous and deliver at a gestational age of 37 weeks or more, while women with known risk factors, such as a multiple pregnancy, previous caesarean delivery or a previous stillbirth, are less likely to give birth unplanned outside an institution (Engjom et al., 2017). These findings indicate that women with a known high‐risk pregnancy receive adequate care. However, another Norwegian study found a 50% increased risk of eclampsia/HEELP in nulliparous women living more than 1 hr away from the hospital, underlining how reduced availability may delay or complicate the identification of obstetric emergencies (Engjom et al., 2018). One Australian study examining the details in 4,096 maternity‐related cases reports various obstetric complications in relation to out‐of‐hospital care, such as postpartum haemorrhage, breech position, cord prolapse, prematurity and neonatal death (McLelland et al., 2018). Even though adverse outcomes might be rare, these studies underline how unidentified obstetric complications could constitute a great risk to women affected. The findings in this study therefore support the conclusion in another Australian study (McLelland et al., 2013); as long as midwives or physicians are not attending unplanned births outside institutions, it is essential that EMTs are adequately educated and equipped to manage labouring women.

## 6 LIMITATIONS

The experiences described in this study are not generalizable to the experiences of all EMTs assisting women giving birth unplanned out‐of‐hospital and the results of this study should therefore be interpreted with caution. While the organization and education is similar in the Nordic setting (Krüger, Skogvoll, Castrén, Kurola, & Lossius, 2010; Langhelle et al., 2004), there is no international standard for EMS. Standards and practices can vary across countries and continents (Lockey, 2009).

## 7 CONCLUSION

Major insufficiencies in the education and training of EMTs advocates for a midwife assisted transport service. There is a need to improve communication between EMTs and midwives and re‐evaluate today's practice, to ensure patient safety for women experiencing unplanned births outside institutions.

## CONFLICT OF INTEREST

We have no conflicts of interest to disclose.

## AUTHOR CONTRIBUTIONS

All authors helped conceptualize ideas, interpret findings and review drafts of the manuscript.

## ETHICAL APPROVAL

This study was approved by the Norwegian Social Science Data Service (NSD, reference number: 54761) and assessed by the Regional Committee for Medical Research Ethics (REC), but considered to be outside the remit of the Act on Medical and Health Research (reference number: 2017/1544). The study was conducted according to the WMA Declaration of Helsinki Principles for Medical Research in Human Subjects (World Medical Association, 2001).
